# Comparison of efficacy and toxicity of bevacizumab, endostar and apatinib in transgenic and human lung cancer xenograftzebrafish model

**DOI:** 10.1038/s41598-018-34030-5

**Published:** 2018-10-26

**Authors:** Yinghua Jin, Lingxiao Wei, Qiuying Jiang, Xiaowei Song, Chong Teng, Chengjuan Fan, Yanju Lv, Ying Liu, Weixi Shen, Li Li, Dayong Huang, Tao Xin

**Affiliations:** Department of Oncology, the Second Affiliated Hospital, Harbin Medical University, Harbin, 150001 P. R. China

## Abstract

The poor prognosis in non-small-cell lung cancer has driven the development of novel targeted therapies. Vascular endothelial growth factor is the most potent force in mediating tumor angiogenesis, and many angiogenesis inhibitors have been developed for oncology treatment. We performed a study to characterize the efficacy, safety and tumor suppression of three lung cancer related anti-angiogenic drugs (bevacizumab, endostar and apatinib) using transgenic zebrafish embryo and human lung cancer xenotransplantation model. All three drugs demonstrated remarkable angiogenesis and tumor inhibition effect in the zebrafish model, within the nonlethal dose range. Endostar and bevacizumab showed competitive anti-tumor efficacy. The anti-tumor performance of apatinib was hamstrung by its elevated toxicity at 35 °C. The addition of pemetrexed to anti-angiogenesis therapy had no obvious additional benefit in tumors.

## Introduction

Non-small-cell lung cancer (NSCLC) remains the most common cause of cancer-related death^1,2^. In many cases, such diseases have reached advanced stages when they are diagnosed, leaving doctors with limited treatment options and leaving patients with poor prognoses. For patients diagnosed with non-oncogenic-driven advanced-stage NSCLC^3,4^, platinum-based doublet chemotherapy is usually recommended, though such standard treatment alone shows a limited survival advantage. Comparatively speaking, some of these patients, who harbor driver gene mutations or have >50% programmed cell death protein ligand 1 (PD-L1) expression, are relatively fortunate to benefit from new strategies, i.e. tyrosine kinase inhibitors (TKIs) and immune checkpoint inhibitors. While giving patients a greater survival advantage, the new strategies, on the other hand, are likely to be thwarted by inevitable emergence of acquired resistance, which result in tumor progression and metastasis^5–9^. Nowadays, for aforementioned patients as well as those who do not possess epidermal growth factor receptor (EGFR) mutation, anaplastic lymphoma kinase (ALK) fusion gene, ROS proto-oncogene 1(ROS1) rearrangement, BRAF V600E mutation or high PD-L1 expression (>50%), anti-angiogenic strategies serve as an alternative or a combination treatment option.

Angiogenesis is essential for tumor growth and metastasis, and anti-angiogenesis is emerging as an effective strategy to treat human cancers^10–12^. In the process of tumor angiogenesis, a number of pathways are involved. Among them, vascular endothelial growth factor (VEGF) signaling pathway has been highly validated and extensively studied^13,14^. It is, therefore, not surprising that VEGF family of proteins and receptors have been closely associated with drug research and development in the field of oncology^15^. Up to now, several anti-VEGF strategies have been developed, including neutralizing antibodies to VEGF or VEGF-receptors (VEGFRs), soluble VEGFR/VEGFR hybrids, and tyrosine kinase inhibitors^16–18^. Recent studies suggest that concurrent therapy using anti-angiogenic and chemotherapeutic agents has achieved promising results. Moreover, the combination of anti-angiogenic therapy and EGFR TKIs also brings hope and benefits to patients with NSCLC^19–21^. Several anti-angiogenic drugs have already been proved to be effective in NSCLC treatment. However, a direct comparison of NSCLC-related angiogenesis inhibitors has yet to be presented.

Bevacizumab, endostar and apatinib are three NSCLC-related anti-angiogenic drugs. Bevacizumab, a recombinant human monoclonal antibody, blocks angiogenesis by inhibiting vascular endothelial growth factor A (VEGF-A)^22^; endostar, a novel recombinant human endostatin, performs its anti-angiogenic action through multiple mechanisms, including targeting endothelial cell VEGFR-2 signaling and osteopontin^23^; apatinib, also known as YN968D1, inhibits angiogenesis by suppressing kinase activities of VEGFR-2, c-kit and c-src^24^. Clinical trials, using such drugs in combination with different toxic drugs and measuring various efficacy endpoints, demonstrated patients with NSCLC could benefit from these drugs. Bevacizumab has been approved as a first-line treatment of advanced nonsquamous NSCLC by the US Food and Drug Administration (FDA), since the ECOG 4599 study demonstrated that bevacizumab in combination with carboplatin/paclitaxel chemotherapy improved overall survival (OS) (12.3 vs. 10.3 months) and progression-free survival (PFS) (6.2 vs. 4.5 months)^22^. When bevacizumab was combined with cisplatin/gemcitabine doublet in metastatic nonsquamous patients, as shown in AVAiL trial, no OS benefit was observed, but a modest value of PFS improvements (6.7 vs 6.1 months)^25^. Endostar, with the approval of China’s State Food and Drug Administration (SFDA), has also been studied in many clinical trials. According to a phase III trial, endostar plus vinorelbine/cisplatin(NP) had better results in response rate (RR) (35.4% vs. 19.5%) and time to progression (TTP) (6.6 vs. 3.7 months) compared with placebo plus NP^26^. A meta-analysis of 15 published clinical studies demonstrated improvements in objective response rate (ORR) (14.7%) and disease control rate (DCR) (13.5%)^27^, when endostar was used in combination with platinum-based chemotherapy (gemcitabine/cisplatin, vinorelbine/cisplatin, paclitaxel/carboplatin, and docetaxel/cisplatin). For apatinib, the improved PFS (4.7 vs. 1.9 months), ORR (12.2% vs. 0%) and DCR (68.9% vs. 24.4%)^28^ revealed the efficacy of such drug in treating metastatic nonsquamous NSCLC, after failure of more than two lines of treatment. In addition to difference in efficacy, these drugs also vary in toxicity. The common side effects related to bevacizumab were proteinuria, hypertension, hemorrhagic events, neutropenia and febrile neutropenia^22,25^. The main adverse effects of endostar were hematological reactions, hepatic toxicity and nausea/vomiting^27^. The most common adverse events in correlation with apatinib were hypertension, proteinuria, and hand/foot syndrome^28^. In view of characteristics of these anti-angiogenic drugs as indicated above, the direct comparison of these drugs will serve as an important reference for future clinical treatment.

In this study, we use zebrafish as an animal model to acquire comparative information from these drugs. Zebrafish, an ideal vertebrate system for cancer and angiogenesis research, helps to present visual images of blood vessel formation and tumor behavior^29^. Furthermore, its embryos exhibit conservation of expression profiles at different levels between fish and human tumor. Importantly, all vertebrates preserve a high extent of molecular conservation involved in angiogenic pathways^30,31^. Considering the increasing proportion of adenocarcinoma among NSCLC histologies (40%)^32^, we chose human alveolar adenocarcinoma cell line A549 for zebrafish xenotransplantation model to evaluate antitumor effect of anti-angiogenic drugs.

In brief, we conducted a direct comparison of three NSCLC-related anti-angiogenic drugs through transgenic zebrafish and zebrafish/tumor xenograft model.

## Results

### Anti-angiogenic activity

**Figure 1:**
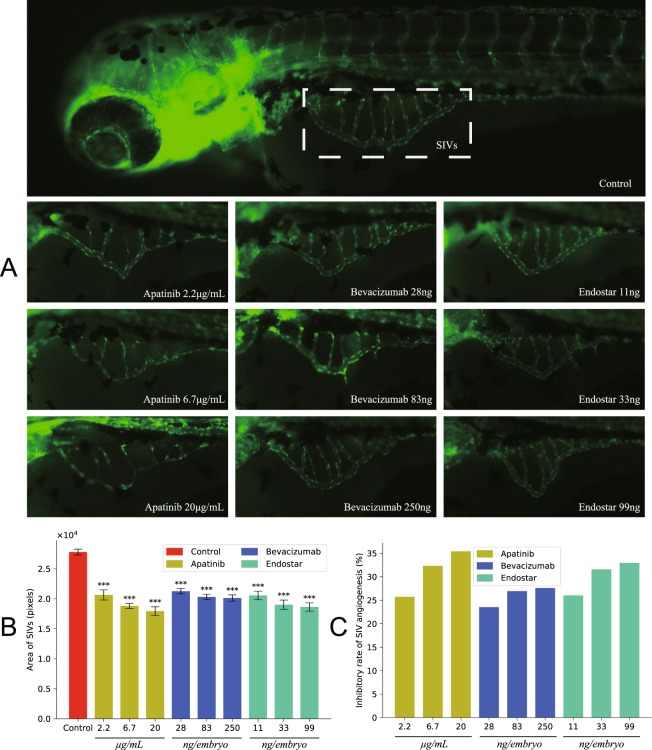
Anti-angiogenic effects after treatment with angiogenesis inhibitors. (**A**) Lateral view of subintestinal vessels (SIVs) in fluorescent transgenic zebrafish embryos at 3dpf that were treated with anti-angiogenic drugs at different concentrations for 24 hours. (**B**) Total area of SIVs (in pixel). (**C**) The inhibitory rate of SIVs angiogenesis is calculated as difference between total area of SIVs in treated and control groups expressed as percentage of total area of SIVs in control zebrafish embryos. The error bars represent ± SEM. P values were determined by one-way ANOVA followed by Dunnett’s test for multiple comparisons to control. ***p < 0.001 indicate statistically significant difference. SEM, standard error of the mean.

The area of subintestinal vessels (SIVs) and antiangiogenic rate resulted from treatment with different concentrations of drugs were shown in Fig. 1. All three drugs showed a dose-dependent anti-angiogenesis effect in SIVs. Apatinib and endostar showed similar results in anti-angiogenic activity, and both were stronger than bevacizumab.

### Anti-cancer effect

**Figure 2:**
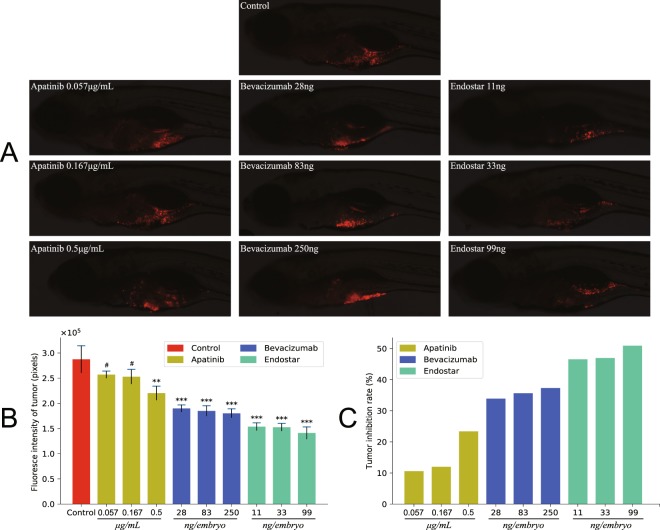
Antitumor effect of anti-angiogenic drugs in the zebrafish embryos. Tumor inhibition effect of three anti-angiogenic drugs in an A549 xenograft zebrafish model. Each compound was evaluated at three different concentrations: bevacizumab (28 ng, 83 ng and 250 ng per embryo), apatinib (0.057 μg/mL, 0.167 μg/mL and 0.5 μg/mL) and endostar (11 ng, 33 ng and 99 ng per embryo). (**A**) Lateral views of CM-Dil stained A549 cells in 5dpf zebrafish embryos treated with compound for 72 hours. (**B**) Fluorescence intensity values of A549 cells in zebrafish larvae. (**C**) Tumor growth inhibition rates is calculated as difference between fluorescence intensities in treated and control groups expressed as percentage of tumor fluorescence intensities in control zebrafish. All quantification data are presented as means ± SEM. P values were calculated with one-way ANOVA followed by Dunnett’s test, **p < 0.01, ***p < 0.001, #p > 0.05 compared to control.

The fluorescence intensities and tumor growth inhibition rates resulted from treatment with different concentrations of drugs were shown in Fig. 2. When apatinib concentration were 0.057 μg/mL and 0.167 μg/mL, both fluorescence intensities and tumor growth inhibition rates were not significantly different (p > 0.05). However, when drug concentration was raised to 0.5 μg/mL, tumor growth inhibition rate was increased to 23%, showing a significant change compared to control group (p < 0.01). Both bevacizumab and endostar could effectively inhibit growth of A549 when a given dosage of drug was injected.

### Anti-tumor activity of anti-angiogenesis alone and in combination with pemetrexed

**Figure 3:**
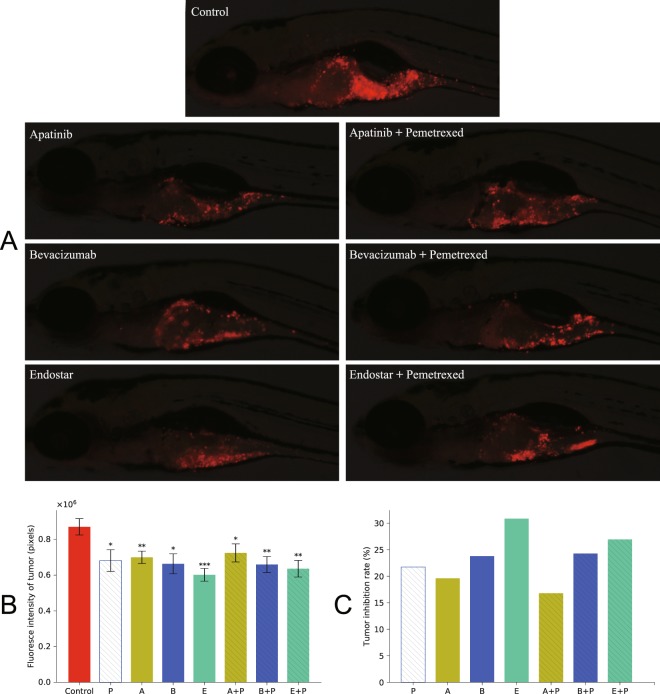
Antitumor activity of anti-angiogenesis alone and in combination with pemetrexed. On 2dpf, CM-Dil labeled A549 xenograft zebrafish embryos were treated with three anti-angiogenesis alone and in combination with pemetrexed for 3 days. Pemetrexed (P) (2 ng per embryo), bevacizumab (B) (28 ng per embryo) and endostar (E) (11 ng per embryo) were injected into embryos and apatinib (A) (0.5 μg/mL) was dissolved in breeding water. (**A**) Fluorescent images showing A549 xenograft zebrafish larvae at 5dpf treated with different inhibitors. (**B**) Quantification of fluorescence intensity of tumor. (**C**) Tumor growth inhibition rates were calculated according to the fluorescence intensities of tumor. Each bar represents the mean ± SEM. One-way ANOVA followed by Dunnett’s test, *p < 0.05, **p < 0.01, ***p < 0.001 when compared with control group.

The fluorescence intensity was measured and tumor growth inhibition rate was calculated after treating zebrafish with different concentrations of anti-angiogenic agents alone or in combination with cytotoxic drugs. As shown in Fig. 3, angiogenesis inhibitors could significantly reduce tumor burden whether as a single agent or in combination with chemotherapy. However, tumor fluorescence intensity and growth inhibition rate were not significantly different whether treating zebrafish with angiogenesis inhibitors alone or in combination with chemotherapy.

### Developmental toxicity and teratogenicity

**Figure 4:**
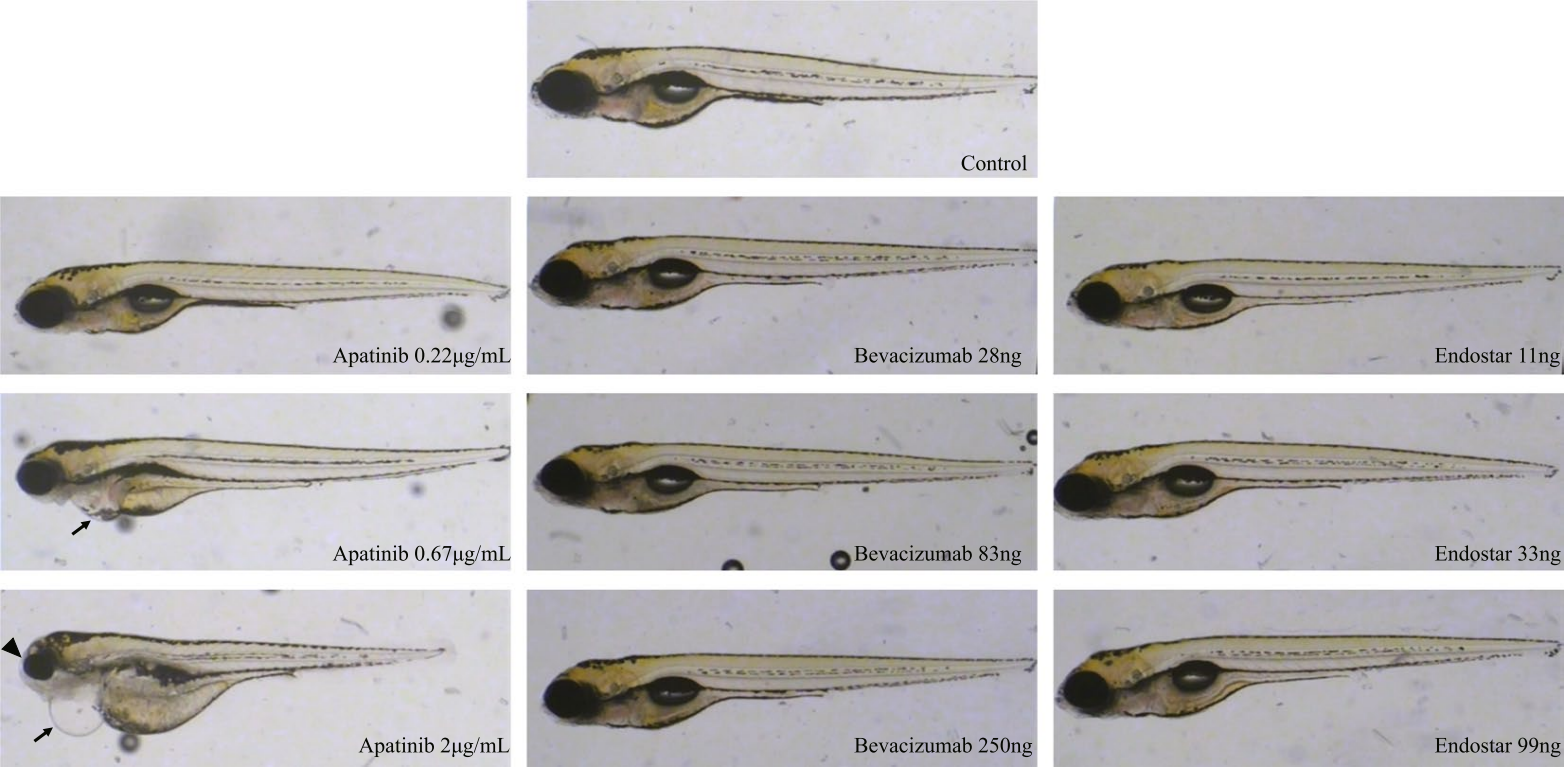
Gross morphological changes in the zebrafish larvae following anti-angiogenic compound exposure at 120 hpf. Wild-type zebrafish were collected at 6 hpf and were treated with bevacizumab (28 ng, 83 ng and 250 ng per embryo), endostar (11 ng, 33 ng and 99 ng per embryo) and apatinib (0.22 μg/mL, 0.67 μg/mL and 2 μg/mL dissolved in breeding water) at 28 °C until 120 hpf. Apatinib (0.67 µg/mL) treatment: pericardial edema (arrow); apatinib (2 µg/mL) treatment: pericardial edema (arrow), decreased eye size (arrowhead) and shortened body axis.

As shown in Fig. 4, all three drugs showed no obvious developmental toxicity or teratogenic effect at 48 hpf and 72 hpf. At 120 hpf, bevacizumab and endostar had no obvious side effect on zebrafish embryos. However, apatinib at all concentration levels (0.22, 0.67 and 2 μg/mL) showed delayed yolk sac absorption, and the incidence was concentration-dependent (50.0%, 63.3% and 100%). When concentration of apatinib was 0.67 µg/mL, ten percent of zebrafish developed pericardial edema. As the concentration of apatinib was raised to 2 µg/mL, morphological abnormality appeared, such as pericardial edema (93.3%), slowed heart rate (60%), decreased eye size (36.7%), and shortened body axis (36.7%).

## Discussion

In this study, we conducted a direct comparison of three lung cancer related anti-angiogenic drugs. The zebrafish model was applied in our experiment. Zebrafish, like other models like chick embryos and rodents, may have limitations when translated to tumor-induced vascular development and cancer research. However, it also presented with advantages. i) The vasculogenesis and angiogenesis of zebrafish are similar at molecular level to other vertebrates. ii) The transparency and ability to survive for days without functioning circulation make it amenable for anti-angiogenesis research^33–35^.

We evaluated the toxicity of three anti-angiogenic agents on zebrafish, and the results demonstrated that only apatinib revealed apparent multi-systemic toxicity at 120 hpf. These toxicity profiles were most cardiovascular-related and consistent with toxicity shown in previous clinical trials (hypertension)^24,36^.

It is worth noting that administration route of apatinib was inconsistent with that of bevacizumab/endostar. Bevacizumab/endostar were intravenously injected, and apatinib was administered in medium. In our experiments, exposure to apatinib with a concentration over 20 μg/mL at 28 °C for 24 hours apparently induced permanent damage to the 48 hpf zebrafish. In order to eliminate the interference of drug toxicity, lower concentrations were used in SIV angiogenesis assay.

In antitumor assay, temperature for incubating xenotransplant zebrafish was increased from 28 °C to 35 °C. Standard temperature for zebrafish incubation was 28 °C, lower than the optimal temperature for culturing xenograft tumor cells (37 °C). As previously reported, increasing incubation temperature to 35 °C brought no apparent gross effect on zebrafish development^37^. Our results demonstrated that, under such temperature, the maximum tolerated dose of apatinib decreased to 0.5 μg/mL, and it was also the maximum concentration for evaluating anticancer activities.

We also investigated long-time incubation toxicity of apatinib. Zebrafish embryos at 6 hpf was incubated, with apatinib for 114 hours, until 120 hpf, and the maximum non-lethal dose noticeably decreased to 2 μg/mL. We suggested that long-time exposure to sustained doses in solution could enhance the developmental toxicity induced by the administration of apatinib.

Since it was reported that the formation of zebrafish vasculature starts at 12 hpf, anti-angiogenesis intervention would be more effective during this period. It might explain the 6 hpf zebrafish embryos were more vulnerable to anti-angiogenic treatment^38^.

In anti-angiogenesis activity assay, all three drugs showed remarkable dose-dependent inhibition effects on zebrafish embryos. For bevacizumab and endostar, our previous study showed that further increased dose than maximum concentration found in this study brought only modest improvement in angiogenesis inhibition efficacy. Given the same test dose, apatinib and endostar had little difference in anti-angiogenic effect, while bevacizumab had a weaker performance. Such disparity might be attributed to different anti-angiogenic mechanisms. Bevacizumab achieved angiogenesis inhibition effect through neutralizing VEGF-A to prevent binding to VEGFR-1, 2^39^. By contrast, both apatinib and endostar were not only selective inhibitors of VEGFR-2, they also preserved other activities against angiogenesis. Specifically, apatinib could suppress the phosphorylation of c-kit and platelet derived growth factor receptors ß (PDGFRß), and suppress kinase activities of Ret, c-kit and c-src^40^. Endostar could inhibit activation of ERK, p38 MAPK, and Akt^41^.

Our study evaluated anti-tumor performance of three angiogenesis inhibitors on A549 xenograft model in the non-lethal dose range. Endostar and bevacizumab had more promising antitumor effects than apatinib. Although apatinib revealed a competitive anti-angiogenic effect, its tumor inhibition rate was minimal. Such discrepancy might be associated with decreased dose. In xenograft experiments, the performance of apatinib was hamstrung by its elevated toxicity at 35 °C. We cannot make the conclusion that apatinib was less effective, since apatinib concentration decreased to one-fortieth of that presented in anti-angiogenic assay.

The antitumor activity of endostar was more preponderant than bevacizumab, while they showed comparative effects in anti-angiogenesis evaluation. Besides starving tumors of oxygen and nutrients, the antitumor activity of angiogenesis inhibitors could also directly induce inhibition effect on tumor cells, which may be ascribed to targeting of VEGFRs expressed on tumor cells^39,42^. A phase II clinical trial argued that bevacizumab could block VEGFR2 activation expressed on breast cancer cells and induced apoptosis in tumor cells^43^. In addition, Wu *et al*.^44^ reported that anti-VEGFR-1 monoclonal antibody could inhibit tumor growth.

Anti-angiogenesis therapies alone could inhibit tumor growth and bring ORR benefit in patients. However, such effect was modest. Moreover, according to preclinical studies, tumor regression following these treatment was found^45–51^. Therefore, combination of anti-angiogenic and cytotoxic agents was expected to achieve better efficacy results. In our study, the addition of pemetrexed to anti-angiogenesis therapy showed no obvious additional efficacy in progressive tumors. Although results of the presented study need to be verified in clinical trials, it still implies that combination of pemetrexed with these three inhibitors did not bring any notable improvements. This lack of joint effect may be ascribed to anti-angiogenic efficacy, which could destroy tumor vessels and hence decrease tumor oxygenation and drug exposure, and finally resulted in antagonism of chemotherapy. It was also argued that anti-angiogenic drugs could normalize tumor vasculature, which could improve tumor blood perfusion and increase tumor exposure to cytotoxic drugs. However, this technique required precise dosage and administration schedule of anti-angiogenic drugs^52^.

In summary, this is the first study directly comparing NSCLC related anti-angiogenic agents (bevacizumab, endostar and apatinib) on various levels, including anti-angiogenic effect, antitumor effect in zebrafish. All three inhibitors demonstrated activities against angiogenesis and cancer development, and acceptable toxicity. The combination of angiogenesis inhibitors with pemetrexed showed no synergistic antitumor effect.

## Methods

### Materials and imaging system

Bevacizumab (Avastin; 25 mg/mL) was purchased from Roche Pharmaceuticals (UK). Pemetrexed disodium (Alimta; 500 mg) was purchased from Eli Lilly and Company (USA). Apatinib and endostar were obtained from Jiangsu Hengrui Medicine Company (China) and Simcere Pharmaceutical Research Company (China) separately. Pemetrexed disodium and apatinib were initially dissolved in dimethyl sulfoxide (DMSO) to stock solutions and further diluted to the desired concentration. Bevacizumab and endostar were diluted by distilled water to a desired concentration before use. Drug treatments were different among three drugs, for pemetrexed, bevacizumab and endostar, 10nL volume of drugs were injected into the embryos, and apatinib was dissolved in breeding water. Images were taken on a Nikon AZ100 fluorescence microscope and analyzed using Nikon NIS-Elements D 3.10 software.

### Zebrafish lines and human lung adenocarcinoma cell line

Zebrafish including the wild-type (AB strain) and the transgenic line fli1:EGFP were provided by Hangzhou Huante biological techonology Co.Ltd (Hangzhou, Zhejiang province, China) and were raised and kept at 28 °C on a 14 h:10 h light-dark cycle under standard circulating water system. The age of embryos was indicated as hours post-fertilization (hpf) and days post-fertilization (dpf). Human lung adenocarcinoma cell line A549 was purchased from the Shanghai Institute of Biological Sciences (Shanghai, China) and maintained in the American Type Culture Collection’s (ATCC) recommended culture medium with 10% of fetal bovine serum.

All experiments conformed to the Association for Assessment and Accreditation of Laboratory Animal Care (AAALAC) guidelines on the ethical use of animals. All experimental protocols were approved by the Animal Care and Use Committee of the Second Affiliated Hospital of Harbin Medical University (Harbin, Heilongjiang, China).

### Zebrafish/tumor xenograft model

After anesthetization, 100 to 200 CM-Dil (a lipophilic fluorescent tracking dye) labelled A549 cells were grafted into yolk sac of each wild-type zebrafish embryos at 48 hpf with a microinjector IM-300 (Narishige, Tokyo, Japan). *In vivo* imaging was performed at 5dpf. After injection, embryos were incubated for 1 h at 28 °C, then at 35 °C.

### Zebrafishsubintestinal vessel angiogenesis assay

The SIV assay used embryos of the transgenic zebrafish line Tg(fli1:EGFP) to evaluate the vasotoxic potential of chemicals. At 2dpf the embryos were collected and randomly divided into ten groups of 30 embryos each, and exposed to different drugs. The zebrafish embryos were maintained in distilled water in 6-well cell culture plates and each well contained 30 embryos. Ten groups were randomized to the following treatment groups: a negative control group; apatinib at concentrations of 2.2 μg/mL, 6.7 μg/mL and 20 μg/mL; bevacizumab at a dose of 28 ng, 83 ng and 250 ng per embryo; endostar at a dose of 11 ng, 33 ng and 99 ng per embryo. Twenty-four hours after treatment, ten embryos were randomly chosen from each group for SIV intensity (S) evaluation. The inhibitory rate of SIV angiogenesis was calculated using the following formula:

1 $${\rm{Inhibition}}\,{\rm{rate}}( \% )=\frac{{\rm{S}}({\rm{negative}}\,{\rm{control}})-{\rm{S}}({\rm{experimental}})}{{\rm{S}}({\rm{negative}}\,{\rm{control}})}\times 100 \% $$

### Xenograft model antitumor assay

Three hundred CM-Dil labeled A549 xenograftzebrafish embryos were randomly chosen and seeded into 10 replicate wells in 6-well cell culture plates (30 embryos per well). Different concentrations of bevacizumab (28 ng, 83 ng and 250 ng per embryo), apatinib (0.057 μg/mL, 0.167 μg/mL and 0.5 μg/mL) and endostar (11 ng, 33 ng and 99 ng per embryo) were applied. After incubation for 3 days at 35 °C, ten out of 30 embryos from each well were randomly chosen and photographed under a fluorescence microscope. The fluoresce intensity of tumor in the embryos (Z) was measured and tumor inhibition rate was calculated according to the following formula:

2 $${\rm{Inhibition}}\,{\rm{rate}}( \% )=\frac{{\rm{Z}}({\rm{negative}}\,{\rm{control}})-{\rm{Z}}({\rm{experimental}})}{{\rm{Z}}({\rm{negative}}\,{\rm{control}})}\times 100 \% $$

### Comparison between antitumor efficacy of anti-angiogenic agents alone and in combination with pemetrexed

On 2dpf, 240 CM-Dil labeled A549 xenograftzebrafish embryos (wild-type AB strain), which were maintained in 6-well cell culture plates (30 embryos each), were distributed into 8 groups according to the following treatment descriptions: pemetrexed (P), apatinib (A), bevacizumab (B), endostar (E), apatinib plus pemetrexed (A+P), bevacizumab plus pemetrexed (B+P), endostar plus pemetrexed (E+P), negative control. As previously described, pemetrexed (2 ng per embryo), bevacizumab (28 ng per embryo) and endostar (11 ng per embryo) were injected into embryos and apatinib (0.5 μg/mL) was dissolved in breeding water. The embryos were then raised at 35 °C for 3 days. At 5dpf, 10 embryos were randomly selected from each well and fluorescence intensity (H) to score for tumor inhibition effect was quantified. The percent tumor growth inhibition was calculated according to the following equation:

3 $${\rm{Inhibition}}\,{\rm{rate}}( \% )=\frac{{\rm{H}}({\rm{negative}}\,{\rm{control}})-{\rm{H}}({\rm{experimental}})}{{\rm{H}}({\rm{negative}}\,{\rm{control}})}\times 100 \% $$

### Zebrafish toxicity assay

The wild-type zebrafish (300 embryos) were collected at 6 hpf and were treated with drugs. All embryos were divided into control and 9 treatment groups. Each group had 30 embryos per test concentration. After treatments, embryos were maintained in an incubator at 28 °C and the result for organ-specific toxic effects was read at 48 hpf, 72 hpf and 120 hpf, respectively. All three anti-angiogenic compounds were evaluated at three different concentrations; bevacizumab and endostar were according to angiogenesis inhibition assay; apatinib was 0.22 μg/mL, 0.67 μg/mL and 2 μg/mL. At 48 and 72 hpf, lethality and developmental disorders were identified by coagulation of the embryo, missing heartbeat, failure to develop somite or a non-detached tail. At 120 hpf, morphological changes in the zebrafish larvae was measured.

### Statistical analysis

Data was subjected to analysis of variance followed by Dunnett’s t-test. A value of p < 0.05 was considered statistically significant.
